# Global South research is critical for understanding brain health, ageing and dementia

**DOI:** 10.1002/ctm2.1486

**Published:** 2023-11-21

**Authors:** Sandra Baez, Suvarna Alladi, Agustin Ibanez

**Affiliations:** ^1^ Global Brain Health Institute (GBHI) Trinity College Dublin (TCD) Dublin Ireland; ^2^ Universidad de los Andes Bogota Colombia; ^3^ Department of Neurology National Institute of Mental Health and Neuro Sciences (NIMHANS) Bangalore India; ^4^ Latin American Brain Health Institute (BrainLat) Universidad Adolfo Ibañez Santiago de Chile Chile; ^5^ Cognitive Neuroscience Center (CNC) Universidad de San Andrés, and CONICET Buenos Aires Argentina; ^6^ Trinity College Dublin (TCD) Dublin Ireland

**Keywords:** ageing, brain health, disparity, diversity

## 1 BRAIN HEALTH, AGEING AND DEMENTIA THROUGH THE LENSES OF DIVERSITY AND DISPARITY

Brain health, ageing and dementia are influenced by various heterogeneous factors, which do not exhibit universal applicability across diverse contexts.^1^, ^2^, ^3^, ^4^, ^5^, ^6^, ^7^, ^8^ Variability in genetics, pathophysiological pathways, clinical presentations, aetiology and exposome is non‐homogeneous across geographical regions.^2^, ^9^ Additionally, unequal epidemiological patterns related to risk and protective factors, social determinants of health (SDH), socioeconomic disparities and healthcare access play important roles in brain health.^1^, ^9^ Thus, brain‐phenotype models developed in more homogenous populations from the Global North may misrepresent the characteristics of more diverse populations.^2^, ^3^ Despite the urgent need to explore regional diversity and provide tailored recommendations, a noticeable research imbalance across regions persists.^3^, ^10^ Paradoxically, most research has been conducted in high‐income settings within the United States and Europe, often overlooking the diverse and non‐stereotypical populations in the Global South.

The impact of high levels of disparities on brain health and ageing in underserved populations is evident across various aspects, including risk factors, brain dynamics, functionality, cognition, biomarkers, neurodegeneration, allostatic overload and access to novel therapeutic targets.^1^, ^6^, ^7^, ^11^, ^12^ Social and environmental disparities, such as low education, elevated poverty rates and heightened exposure to adversity, increase the prevalence of chronic conditions associated with brain health and dementia risks.^13^ For instance, metabolic disorders such as Type‐2 diabetes mellitus, cardiovascular disease and cerebrovascular disease are more prevalent among underserved populations.^13^, ^14^ We recently found^1^ that health disparities and SDH, such as education, cardiometabolic conditions and social isolation, severely influence the healthy ageing of Latin American populations, impacting cognition and functional ability more than traditional factors like age and gender. This heterogeneity in risk factors was particularly pronounced in lower‐ to middle‐income countries (Colombia and Ecuador). In addition, while educational attainment predicts cognitive performance in older adults from homogeneous populations,^13^, ^15^ this relationship weakens among heterogeneous groups due to disparities in access to high‐quality education. Altogether, these findings emphasise the significant influence of disparities and region‐specific factors on brain health and ageing. These effects seem to be more accentuated across Global South populations, evidencing the inadequacy of a one‐size‐fits‐all approach.

Similarly, population diversity and disparity have a strong effect on dementia.^8^, ^11^, ^13^, ^16^ North Africa/Middle East (8.7%) and Latin America (8.4%) exhibit the current highest dementia prevalence, with Central Europe having the lowest one (4.7%).^17^ The larger prevalence in the Global South is associated with health and socioeconomic disparities.^8^, ^11^ Diversity and disparities are also evident in the projected increases in the number of people living with dementia in 2050, with the most significant percentage changes in North Africa and the Middle East (367%) and the smallest in Western Europe (74%).^17^ Additionally, dementia genetic risk factors, such as the apolipoprotein (APOE) 4 allele, the most significant ged8netic risk factor for Alzheimer's disease (AD), seem to differ across populations. This allele is more frequent among individuals with African ancestry but has an attenuated association with AD risk, compared to those of European ancestry.^18^ The influence of diversity and disparity is further evident in marked inequalities in care‐seeking and dementia diagnosis among the Global South. For example, in many low‐ and middle‐income countries, the diagnosis of dementia often occurs at a more advanced stage due to low societal awareness, compared to Western countries.^2^ The caregiver burden is increased in Latin America, compared to other regions, due to structural adversities in health and social factors.^19^ Moreover, most neuropsychological tests used for dementia diagnosis have been developed for educated, predominantly English‐speaking Western populations and may not be suitable for use in other cultures.^2^, ^16^ Taken together, this evidence highlights that interactions between heterogenous SDH, genetics, biological, exposome and psychosocial factors significantly influence dementia phenotypes, prevalence, risk and diagnosis. To systematically investigate these interactions, it is imperative to include greater diversity from non‐stereotypical populations.

**FIGURE 1:**
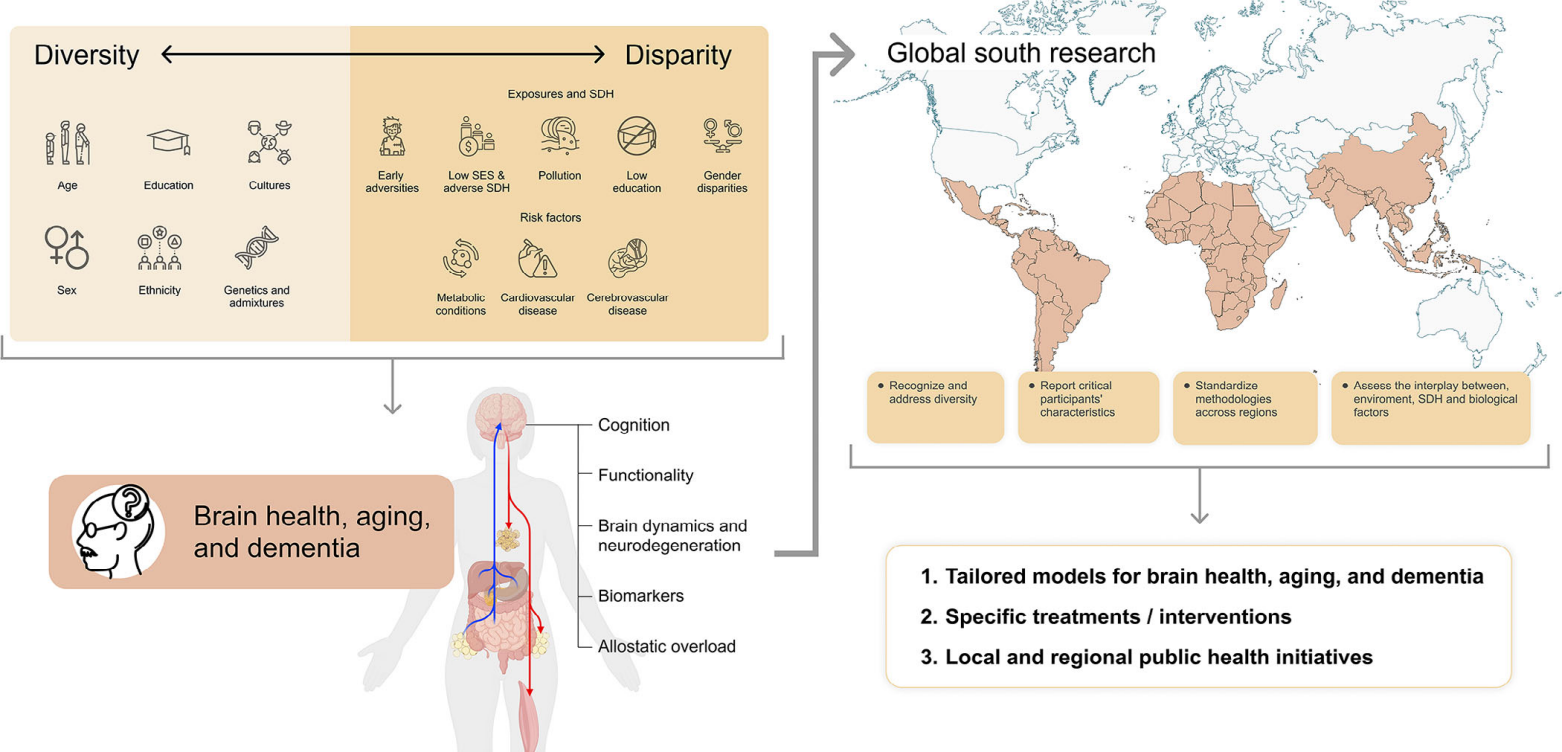
Diversity and disparity impact brain health, ageing and dementia. The left panel illustrates the interconnection between diversity and disparity and their impact on various dimensions of brain health, ageing and dementia (i.e., cognition, functionality, brain dynamics and neurodegeneration, biomarkers and allostatic overload). The upper right diagram highlights critical recommendations for addressing diversity and disparity and further understands their effects on the mentioned multidimensional factors in non‐stereotypical populations from the Global South. In the lower right corner, we outline the main expected outcomes of the proposed Global South research approach. SDH, social determinants of health; SES, socioeconomic status.

Despite the acknowledged influence of diversity and disparity on brain health, ageing and dementia,^6^, ^7^, ^11^, ^12^ more comprehensive assessments of non‐stereotypical populations from the Global South are required (See Figure 1). Future methodological strategies should recognise and embrace the diversity within each country and region to address structural inequities.^1^ In future endeavours, it is imperative to prioritise the inclusion of underrepresented older adult groups, improve the documentation of critical participant characteristics and assess the proportional representation of different racial, ethnic and minority groups.^18^ Future research needs to integrate sources of population‐level and clinical data and utilise robust methods to address heterogeneity driven by diverse genetic, health, biological, functional, social, behavioral, financial and neighborhood influences on brain health, ageing and dementia in the Global South.^9^, ^20^

Efforts to harmonise and standardise methodologies across regions in the Global South are urgently needed. Initiatives to harmonise clinical research methods have been primarily focused on Caucasian populations, neglecting underrepresented populations.^2^ Incorporating genetic and imaging methods alongside locally validated clinical protocols is essential to address diversity effectively.^2^ Also, harmonisation frameworks across various disciplines, including epidemiology, clinical research, basic neuroscience and social sciences, are critically needed to provide a comprehensive understanding of the interplay between environmental and biological factors.^4^, ^5^ Identifying the most effective interventions for dementia in diverse sociocultural contexts of the Global South is crucial. This approach can provide more robust predictive models for brain health and ageing, as well as potential treatments for dementia tailored to the specific needs of diverse populations. These models can serve as valuable guides for local and regional public health initiatives.^1^ To develop customised preventive measures, public health and clinical sciences leaders should recognise the complex interplay of disparity‐related factors, including individual health indicators and SDH from a local setting.^4^, ^5^ This tailored approach represents the foundation for shaping policies that yield synergistic benefits across various aspects of brain health, ageing and dementia (See Figure 1)

## CONFLICT OF INTEREST STATEMENT

The authors declare no conflicts of interest.
